# Medication Deprescribing in Patients Receiving Hemodialysis: A Prospective Controlled Quality Improvement Study

**DOI:** 10.1016/j.xkme.2024.100810

**Published:** 2024-03-20

**Authors:** Émilie Bortolussi-Courval, Tiina Podymow, Marisa Battistella, Emilie Trinh, Thomas A. Mavrakanas, Lisa McCarthy, Joseph Moryousef, Ryan Hanula, Jean-François Huon, Rita Suri, Todd C. Lee, Emily G. McDonald

**Affiliations:** 1 Division of Experimental Medicine, Faculty of Medicine and Health Sciences, McGill University, Montreal, Quebec, Canada; 2 Division of Nephrology, Department of Medicine, McGill University Health Centre, Montreal, Quebec, Canada; 3 Leslie Dan Faculty of Pharmacy, University of Toronto, Toronto, Ontario, Canada; 4 Faculty of Medicine and Health Sciences, McGill University, Montreal, Quebec, Canada; 5 Department of Medicine, McGill University Health Centre, Montreal, Quebec, Canada; 6 Division of Pharmacy, Nantes University Health Centre, Nantes University, Nantes, France; 7 Division of Infectious Disease, Department of Medicine, McGill University Health Centre, Montreal, Quebec, Canada; 8 Clinical Practice Assessment Unit, Department of Medicine, McGill University Health Centre, Montreal, Quebec, Canada; 9 Division of General Internal Medicine, Department of Medicine, McGill University Health Centre, Montreal, Quebec, Canada

**Keywords:** Chronic kidney disease, deprescribing, electronic decision support, hemodialysis, polypharmacy

## Abstract

**Rationale & Objective:**

Patients treated with dialysis are commonly prescribed multiple medications (polypharmacy), including some potentially inappropriate medications (PIMs). PIMs are associated with an increased risk of medication harm (eg, falls, fractures, hospitalization). Deprescribing is a solution that proposes to stop, reduce, or switch medications to a safer alternative. Although deprescribing pairs well with routine medication reviews, it can be complex and time-consuming. Whether clinical decision support improves the process and increases deprescribing for patients treated with dialysis is unknown. This study aimed to test the efficacy of the clinical decision support software MedSafer at increasing deprescribing for patients treated with dialysis.

**Study Design:**

Prospective controlled quality improvement study with a contemporaneous control.

**Setting & Participants:**

Patients prescribed ≥5 medications in 2 outpatient dialysis units in Montréal, Canada.

**Exposures:**

Patient health data from the electronic medical record were input into the MedSafer web-based portal to generate reports listing candidate PIMs for deprescribing. At the time of a planned biannual medication review (usual care), treating nephrologists in the intervention unit additionally received deprescribing reports, and patients received EMPOWER brochures containing safety information on PIMs they were prescribed. In the control unit, patients received usual care alone.

**Analytical Approach:**

The proportion of patients with ≥1 PIMs deprescribed was compared between the intervention and control units following a planned medication review to determine the effect of using MedSafer. The absolute risk difference with 95% CI and number needed to treat were calculated.

**Outcomes:**

The primary outcome was the proportion of patients with one or more PIMs deprescribed. Secondary outcomes include the reduction in the mean number of prescribed drugs and PIMs from baseline.

**Results:**

In total, 195 patients were included (127, control unit; 68, intervention unit); the mean age was 64.8 ± 15.9 (SD), and 36.9% were women. The proportion of patients with ≥1 PIMs deprescribed in the control unit was 3.1% (4/127) vs 39.7% (27/68) in the intervention unit (absolute risk difference, 36.6%; 95% CI, 24.5%-48.6%; *P* < 0.0001; number needed to treat = 3).

**Limitations:**

This was a single-center nonrandomized study with a type 1 error risk. Deprescribing durability was not assessed, and the study was not powered to reduce adverse drug events.

**Conclusions:**

Deprescribing clinical decision support and patient EMPOWER brochures provided during medication reviews could be an effective and scalable intervention to address PIMs in the dialysis population. A confirmatory randomized controlled trial is needed.

## Plain-Language Summary

Patients treated with dialysis are commonly prescribed multiple medications, some of which are potentially inappropriate medications (PIMs). PIMs can increase a patient’s pill burden and are associated with an increased risk of harm (some examples include falls, fractures, and hospitalization). Deprescribing is a proposed solution that aims to highlight medications that can be stopped, reduced, or switched to a safer option, under supervision of a health care provider. We aimed to determine if a quality improvement intervention in the dialysis unit could increase deprescribing compared to usual care. The study took place in 2 outpatient hemodialysis units where usual care involves nurses and nephrologists performing medication reviews twice a year. The intervention was a deprescribing report that was generated with the help of a software tool called MedSafer, along with brochures for patients with information on PIMs they were taking. In the intervention unit, we increased the number of patients who had a medication safely deprescribed by 36.6% more than on the control unit. Although the study was small, a future larger study in dialysis patients might show that a computer software such as MedSafer can prevent harmful complications from taking too many medications.

Editorial, ●●●

The burden of polypharmacy (taking multiple medications) in patients who receive dialysis is substantial: more than 90% of patients take 5 or more medications.^1^ The majority are important to modify cardiovascular risk and to assist in maintenance of calcium and phosphate balance. However, in addition to indicated and beneficial medications, many are also potentially inappropriate.^2^^,^^3^ Potentially inappropriate medications (PIMs) may have limited benefit, can increase a patient’s pill burden,^4^ and are associated with an increased risk of adverse drug events (ADEs).^5^^,^^6^ An ADE is an umbrella term for harm arising during medication therapy^7^; examples include falls,^8^ fractures,^9^ and cognitive impairment.^10^ An ADE can occur from an individual PIM or through drug-drug or drug-condition interactions.^11^ It is increasingly recognized that polypharmacy and ADEs are common, costly, and harmful to patients and the health care system, contributing to preventable emergency room visits, hospital admissions, premature loss of autonomy, and death.^9^^,^^12^ Given that 93% of patients treated with dialysis are estimated to be receiving one or more PIMs,^1^ pragmatic, scalable deprescribing interventions to reduce medication burden in this patient population are needed.^13^, ^14^, ^15^

Deprescribing is defined as the “process of withdrawal of an inappropriate medication, supervised by a health care professional, with the goal of managing polypharmacy and improving outcomes.”^16^ It has been shown to reduce the number of prescribed drugs and, in some studies, reduce the risk of falls, hospitalization, and mortality.^17^ MedSafer is an electronic decision support tool for deprescribing that has been shown to increase deprescribing for hospitalized older adults and for people residing in long-term care.^1^^,^^11^^,^^18^^,^^19^ In a cluster randomized controlled trial (RCT) of 5,698 hospitalized older adults, when compared to usual care, paired receipt of deprescribing reports and relevant patient information EMPOWER brochures increased the proportion of patients with one or more PIMs deprescribed by 22.2% (95% confidence interval [CI], 16.9%-27.4%).^18^ MedSafer is a Canadian-built software that cross references a person’s usual medication list and their medical diagnoses with a curated ruleset of evidence-informed deprescribing guidelines (Box 1).^18^^,^^20^, ^21^, ^22^ Reports generated through the software identify candidate PIMs for deprescribing, or so-called deprescribing opportunities, ordered by level of potential harm, along with tapering regimens for drugs considered at risk for withdrawal reactions.^20^, ^21^, ^22^, ^23^ This RCT included 140 patients who were receiving maintenance hemodialysis (∼2.5% of the overall study population); in the dialysis subgroup, the proportion of patients with one or more PIMs deprescribed increased by 9.4% (95% CI, 1.3%-17.6%)^1^ with the intervention, which was lower than the rate of 22.2% observed in the general study population.^18^ We hypothesized that the lower rate of deprescribing was related to the complexity of medical admissions for patients receiving dialysis and, perhaps, due to a lack of dialysis-specific deprescribing rules.

Box 1 **MedSafer: the electronic deprescribing clinical decision support tool**

Deprescribing guidance

MedSafer identifies deprescribing opportunities by electronically cross-referencing a person’s usual medication list and medical comorbid conditions with a curated ruleset containing evidence-based deprescribing guidelines (based on criteria from the American Geriatrics Society,^20^ STOPP,^21^and Choosing Wisely^22^). MedSafer stratifies deprescribing opportunities into high risk, intermediate risk, or little added value categories. High risk implies there is an elevated risk of developing adverse drug events, intermediate risk implies that the harms must be weighed against the benefits of the drug, and drugs of little added value simply increase the pill burden of a patient or have evidence demonstrating no effect.^18^

After the RCT was published, Lefebvre et al^13^ proposed additional dialysis-specific deprescribing algorithms as part of a quality improvement initiative. We set out to integrate these algorithms into the MedSafer software and to study the efficacy of clinical decision support for deprescribing in the outpatient dialysis unit setting.

## Methods

### Design and Setting

This prospective, controlled, quality improvement study is reported using the SQUIRE guidelines.^24^ It was prospectively registered in ClinicalTrials.gov (NCT05585268).^25^ The study took place between September and December 2022 in the 2 largest (of 3) outpatient hemodialysis units at the McGill University Health Centre in Montreal, Quebec, Canada. The detailed protocol for this study was published previously.^26^ The intervention unit was the Lachine Hospital dialysis unit, and the control unit was the Montreal General Hospital dialysis unit. The physician-champion randomly assigned these units. Because this was largely an educational quality improvement intervention directed at the treating nephrologists, randomizing at the level of the individual patient would not have been feasible because this would have contaminated the intervention.^27^ The intervention unit dialyzes approximately 80-90 patients per week and the control unit approximately 150-155. Patients visit the units thrice weekly on average. Both units use the electronic medical record Renal Insight (Constellation Kidney Group, previously known as NephroCare)^28^ to store clinical data. Renal Insight contains clinical data such as medical diagnoses, home medications, and laboratory values and is bidirectionally integrated with the hospital’s main electronic medical record (OACIS, Telus Health).^29^ Our a priori sample size calculation suggested we would have 80% power to demonstrate at least a 15% increase in the proportion of patients with 1 or more PIMs deprescribed.^26^

We paired the intervention as part of usual workflow known as “medication reconciliation.”^30^ This is an interdisciplinary clinical activity performed biannually in our hemodialysis units in the Spring and Fall and within 1 week following hospital discharge (Fig S1). The usual reconciliation process occurs as follows: a dialysis nurse reviews the list of usual home medications and compares this with the medication list provided by the community pharmacy, noting any discrepancies. Afterward, the treating nephrologist and nurse jointly review these data and perform necessary adjustments. This process aims to confirm appropriate dosing and avoid duplications, omissions, or errors. There is no clinical pharmacist in either unit. Deprescribing may occur, but it is not protocolized and depends on the nephrologist’s clinical judgment.

### The Intervention

MedSafer stratifies deprescribing opportunities (eg, PIMs that have the potential to be deprescribed) into categories of high risk, intermediate risk, or medications of little added value, informed by indications based on patient comorbid conditions and past medical history.^18^ High risk equates to an elevated risk of developing an ADE, intermediate risk medications have harms that must be weighed against the benefits, and drugs of little added value superfluously increase the pill burden of a patient or have evidence demonstrating no effect.^19^ Examples of a typical deprescribing report can be found in the Supplementary Material. Patients also received deprescribing EMPOWER brochures for select classes of PIMs (eg, opioids, gabapentinoids, sedative hypnotics).^31^ These brochures contain nonpharmacologic alternatives and information about the potential harms of the medication class (see Item S2 for an example). This multimodal approach (providing deprescribing reports to clinicians and brochures to patients) was successfully implemented in a prior large RCT with MedSafer.^18^

### Planning the Intervention

Both units had a scheduled medication reconciliation planned for Fall 2022 (September-December 2022), during which time we implemented and studied this quality improvement intervention. The physicians taking part in the study attended solely in one of the 2 units and did not cross over between sites. The control unit performed medication reconciliation as usual care, without the provision of deprescribing reports to nephrologists or brochures to patients. In the intervention unit, an introductory email was sent to the attending nephrologists containing the overview of the study and how the MedSafer reports and deprescribing brochures would integrate with the existing medication reconciliation workflow of 10-15 medication reconciliations per week. Nephrologists were also provided with a sample MedSafer report to familiarize themselves with the output. A nephrologist champion (TP), for our study purposes defined as a physician facilitating the change necessary to implement a new electronic health information technology,^32^ was available to answer inquiries.

Reports were generated in advance before the patient’s scheduled medication reconciliation and were alphabetically stored in a binder on the intervention unit to be used as part of the exercise. The nephrologist would notify the study team of upcoming medication reconciliations and they would be provided the patient’s documentation package from the binder to review. Five sequential Plan-Do-Study-Act (PDSA) cycles were subsequently used as an implementation strategy to achieve the aims of the project in the intervention unit (Fig S2).^33^^,^^34^ Each PDSA cycle was preceded by a system analysis that identified specific barriers inhibiting the success of the workflow.^33^

## Outcomes

At the end of the intervention period for both units (December 2022), the medication reconciliation notes were reviewed to identify any deprescribing of PIMs (medications flagged by the MedSafer deprescribing report).

The primary outcome was a process measure: the proportion of patients with one or more PIMs deprescribed. Subgroup analyses by age category (<65 vs >65) were prespecified. Deprescribing was defined as any PIM that was either stopped, deliberately reduced, or tapered.^18^^,^^19^

Key secondary outcomes included the reduction in the mean number of prescribed drugs and PIMs from baseline. Although this study was not sufficiently powered to have an effect on ADEs (including adverse drug withdrawal events), we nonetheless collected 2 counterbalancing indicators of harm for descriptive purposes: gastrointestinal bleeds (GIBs) within 3 months following the intervention and death following the intervention (see Item S1). GIBs were selected as a counterbalancing measure (an approach used in quality improvement studies)^35^ because a prior, uncontrolled, observational study of deprescribing proton pump inhibitors (PPIs) in patients receiving dialysis found that 2 of 29 patients (7%) had a GIB within 2-4 weeks of having the PPI deprescribed.^36^ Implementation barriers and facilitators were collected from semistructured interviews with nephrologists (reported separately).

### Recruitment of Patients

For the purposes of analysis, only the initial closed cohort was included in the study. This cohort was comprised patients receiving maintenance hemodialysis (>3 months). New patients initiated on maintenance hemodialysis during the study, transplanted patients, and those hospitalized, transferred to another dialysis unit, or who died before their regularly scheduled medication reconciliation were excluded from the final analysis.

### Ethics

The McGill University Health Centre Director of Professional Services approved the plan for the quality improvement activity, granted access to medical charts, and provided a waiver of consent for the intervention.

### Data Collection

From Renal Insight, the study lead extracted, for all patients in the study, medical conditions (diagnoses), usual home medications (from the best possible medication history informed by the community pharmacy’s list and the electronic medical record’s list of medications), and recent glycated hemoglobin. This data were input into the MedSafer web-based portal, and opportunities for deprescribing were assessed. Reports were only provided to nephrologists for patients in the intervention unit.

### Data Analysis

Descriptive statistics were used to compare baseline health characteristics between patients. χ^2^ and Fisher exact tests were used to compare categorical differences. Wilcoxon rank-sum tests and *t* tests were used to compare medians and means between groups. For the primary outcome, we used a 2-sample test of proportions with 95% CIs. For the number of drugs before and after medication reconciliation, we used logistic regression, adjusting for the presence of the intervention and the number of baseline drugs. Covariates were selected a priori based on known potential confounders and pragmatically, based on the availability of data in the electronic medical record. All statistical comparisons used a 2-sided α of 0.05 as significant with no adjustment for multiplicity of testing.

## Results

### PDSA Iterations

In PDSA cycle 1, to improve efficiency, the study lead prepared and ordered packages for the intervention alphabetically (Fig S2). In PDSA cycle 2, to reduce the burden on the care team, we made it explicit that only patients with select PIMs deprescribed needed to receive EMPOWER brochures. During PDSA cycle 3, to facilitate data extraction, the keyword “MedSafer” was entered into their progress note to document the intervention had taken place. During PDSA cycle 4, to improve efficiency, the study lead emailed the attending nephrologist the list of patients who were due for medication reconciliation 1 business day before the start of their rotation. During PDSA cycle 5, to improve efficiency, the list of PIMs for each patient was provided to the nephrologist before rounding.

### Population

**Figure 1:**
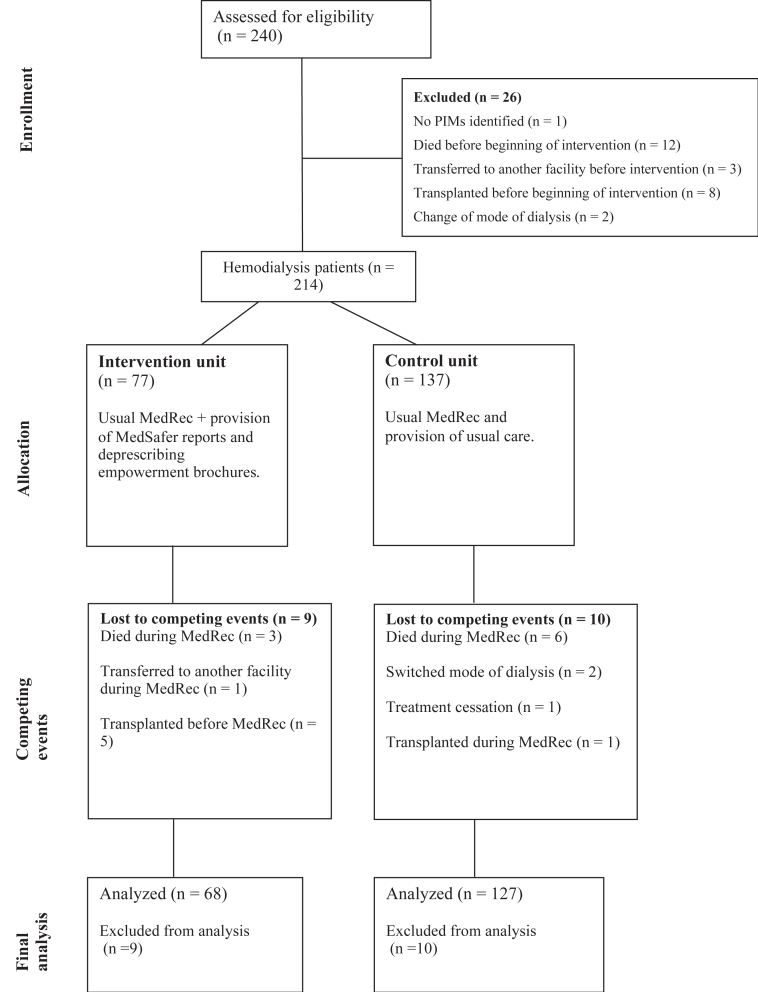
Consort flow diagram of hemodialysis patients assessed for study inclusion.

Initially, 240 patients were assessed for eligibility (Fig 1), and 26 (10.8%) were excluded before beginning the study: 18 from the control unit and 8 from the intervention unit. Twelve died before the beginning of the intervention, 8 were transplanted before the start of the study, 3 were transferred to another facility, 2 changed mode of dialysis, and 1 patient had no PIMs identified. During the study, an additional 10 patients were excluded in the control unit and 9 in the intervention unit; these patients were enrolled in the study but did not receive any medication reconciliation because of these events (Fig 1).

Ultimately, 195 patients were included in the final analysis (127 in the control and 68 in the intervention unit). The mean age was 64.8 ± 15.9 (SD) and 36.9% were women (Table 1). The 3 most prevalent comorbid conditions were hypertension (n = 173, 88.7%), dyslipidemia (n = 124, 63.6%), and diabetes (n = 114, 58.5%). Intervention and control unit patients were similar with respect to common medical conditions, except for diabetic neuropathy, orthostatic hypotension, and gastroesophageal reflux disorder, which were more prevalent in the intervention unit (Table 1). Patients were prescribed a mean of 15.3 (5.3) medications in the control unit and 14.6 (4.7) medications in the intervention unit (*P* = 0.33) and a median of 4 PIMs (interquartile range, 3-6) in both the control and intervention units (*P* = 0.5).

**Table 1:** Baseline Patient Characteristics

Characteristic	Control (N = 127)	Intervention (N = 68)	*P*
Demographic information			
Age, mean (SD)	64.8 (16.9)	64.7 (13.8)	0.9
Female sex	50 (39.4%)	22 (32.4%)	0.4
Medications			
Number of medications, mean (SD)	15.3 (5.3)	14.6 (4.7)	0.3
Number of PIMs identified, median (IQR)	4 (3-6)	4 (3-6)	0.5
Comorbid condition			
Diabetes	79 (62.2%)	35 (51.5%)	0.2
Diabetic neuropathy	64/79 (81.0%)	35/35 (100%)	0.006
Hypertension	112 (88.2%)	61 (89.7%)	0.8
Dyslipidemia	82 (64.6%)	42 (61.8%)	0.7
Ischemic heart disease	36 (28.3%)	17 (25%)	0.6
Heart failure	35 (27.6%)	22 (32.4%)	0.5
Atrial fibrillation	16 (12.6%)	8 (11.8%)	0.9
Valvular heart disease	11 (8.7%)	8 (11.8%)	0.5
History of ischemic stroke	11 (8.7%)	9 (13.2%)	0.3
History of venous thromboembolism	10 (7.9%)	7 (10.3%)	0.6
COPD	12 (9.4%)	2 (2.9%)	0.09
Asthma	9 (7.1%)	5 (7.4%)	0.9
Orthostatic hypotension	3 (2.4%)	15 (22.1%)	<0.001
Gastroesophageal reflux disease	5 (3.9%)	13 (19.1%)	<0.001
History of gastrointestinal bleed	11 (8.7%)	7 (10.3%)	0.7
Constipation	33 (26%)	20 (29.4%)	0.6
Solid organ cancer	23 (18.1%)	20 (29.4%)	0.07
Psychiatric disorder^a^	25 (19.7%)	11 (16.2%)	0.6
Parkinson disease	3 (2.4%)	0 (0%)	0.2

*Note*: Values are shown as n (%), unless otherwise indicated.

Abbreviations: COPD, chronic obstructive pulmonary disease; IQR, interquartile range; PIM, potentially inappropriate medication.

a Substance use disorder, major depressive disorder, bipolar affective disorder, schizophrenia.

### Primary Outcome

**Figure 2:**
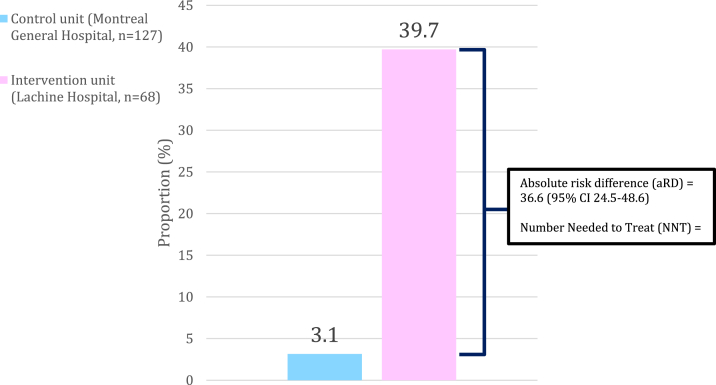
Proportion of patients with 1 or more PIMs deprescribed by intervention status. PIM, potentially inappropriate medication.

The proportion of patients with one or more PIMs deprescribed in the control unit was 3.1% (4/127) compared with 39.7% (27/68) in the intervention unit for an absolute increase of 36.6% (95% CI, 24.5%-48.6%; *P* < 0.0001; Fig 2). The number needed to treat for deprescribing was 3. The subgroup analysis stratified by age showed efficacy in both patients above and below 65 years of age (Fig S3). Of the 45 PIMs deprescribed in both units, 5 (11.1%) were from patients in the control unit and 40 (88.9%) from patients in the intervention unit. From both units, 11 PIMs (24.4%) were high-risk (eg, a sedative hypnotic), 22 (48.8%) were intermediate-risk (eg, long-term use of a non–evidence-based PPI), and 12 (26.6%) were low-risk (eg, docusate).

### Secondary Outcomes

Following medication reconciliation, the mean ± SD number of medications prescribed was 15.3 ± 5.3 in the control unit and 14.0 ± 4.6 in the intervention unit. The linear regression model (Table 2) showed that, after adjusting for the intervention status of the patients and their baseline number of medications, the difference in the mean number of medications prescribed after the intervention decreased by −0.54 medications per patient (95% CI, −0.69 to −0.39; *P* < 0.0001). In the intervention unit, 11 of 38 (29%) of the deprescribing opportunities related to the newly integrated dialysis-specific rules.

**Table 2:** Outcomes

Characteristic	Control (N = 127)	Intervention (N = 68)	*P*
Number of patients with ≥1 PIMs deprescribed (n, %)	4 (3.1%)	27 (39.7%)	<0.0001
Absolute risk difference (RD) of deprescribing	RD 36.6 (95% CI, 24.5-48.6)		
Number Needed to Treat (NNT)	3		
Mean estimated change in total drugs (95% CI)	REF	−0.54 (95%CI, −0.69 to −0.39)	<0.0001

Abbreviations: CI, confidence interval; PIM, potentially inappropriate medication; REF, referent comparison group.

### Counterbalancing Outcomes

Following medication reconciliation, 2 patients died in the control unit and 1 patient died in the intervention unit. None of the deaths were related to deprescribing. Five patients in the control unit, and 2 in the intervention unit had a GIB (Table 3). None of the GIBs were related to PPI deprescribing. In the control unit, 4 of 5 patients had a GIB despite being on a PPI. In the intervention unit, at the time of the GIB, 1 patient was actively prescribed a PPI, and the other was never prescribed a PPI.

**Table 3:** Counterbalancing Measure of Harm: Gastrointestinal Bleeds

Bleeding Episode^a^	Allocation	Did GIB Lead to Death?	Proton Pump Inhibitor Status at Time of GIB	Anticoagulants Prescribed at Time of GIB
Patient 1	Control	Yes	Active prescription	—
Patient 2	Control	No	—	—
Patient 3	Control	No	Active prescription	Aspirin 80 mg daily
Patient 4	Control	No	—	—
Patient 5	Control	No	—	—
Patient 6	Intervention	No	Active prescription	—
Patient 7	Intervention	No	Active prescription	Aspirin 80 mg daily

Abbreviation: GIB, gastrointestinal bleed.

a During the study and for 3 months after intervention.

## Discussion

This is one of the first trials to increase PIM deprescribing among patients on hemodialysis, which we accomplished with a number needed to treat of 3, compared with usual care. Patients on hemodialysis are prescribed multiple medications; in our study, patients took an average of 15 medications. Identifying PIMs in a list of over a dozen medications can be laborious and time-consuming for clinicians. We aimed to make the process more accessible for nephrologists by leveraging an existing workflow, medication reconciliation, as the opportunity for medication “rationalization,” using electronic decision support. We noted higher rates of deprescribing in this study compared with our RCT (number needed to treat of 4-5), possibly because of the addition of hemodialysis-specific deprescribing indications. Other reasons can be attributed to the single-center nature of this study versus the multicentered trial, to differences between the acute care setting and the dialysis unit, or to differences between nephrologists vs other subspecialists attending the inpatient units of the RCT.

The use of the MedSafer technology to generate a deprescribing report addressed 2 key barriers to deprescribing: patient medical complexity and the time-consuming nature of the process.^1^^,^^2^^,^^11^^,^^18^^,^^37^, ^38^, ^39^, ^40^, ^41^, ^42^, ^43^ These barriers are addressed by leveraging technology, in this case the backend of the software, which contains hundreds of algorithms with opportunities for deprescribing. It further provides the clinical and scientific rationale for deprescribing, along with tapering instructions (when needed), at the point of care. The aforementioned barriers are particularly true for patients with end-stage kidney disease who have multiple coexisting medical conditions and are often treated with 12-15 medications.^1^^,^^18^ To our knowledge, this is the first controlled study to test the newly developed dialysis-specific deprescribing guidelines by Lefebvre et al.^13^ Our results align with 2 small noncomparative studies that previously evaluated the efficacy of providing dialysis-specific deprescribing recommendations^15^^,^^44^; one study of 5 dialysis-specific medication class recommendations deprescribed 78% of PIMs identified.^15^ Another study implemented 8 dialysis-specific deprescribing algorithms and managed to deprescribe 35 of 59 (59.3%) PIMs, and 27 of 35 (77%) of these remained deprescribed at 16 weeks following the intervention.^44^

Our studies differed in our use of a contemporaneous control unit to observe differences with usual care. Furthermore, our reports contained both dialysis-specific and general deprescribing opportunities from multiple sources.^13^^,^^20^, ^21^, ^22^ We also provided EMPOWER brochures to augment the intervention and engage patients. Of note, the opportunities we flagged often contained deprescribing opportunities typically geared toward older adults. However, in a prespecified subgroup analysis, the intervention was equally effective in both younger and older adults. Other strengths included leveraging Renal Insight, integrating with the existing medication reconciliation workflow, and use of a previously tested software to facilitate deprescribing decision support. We also deployed PDSA cycles to iteratively improve the process.

There are several limitations to this study worth discussing. First, we implemented 2 interventions simultaneously (eg, decision support and patient brochures); consequently, it was not possible to quantify the individual effect of each intervention. Both interventions have been shown to independently increase deprescribing, and we used the same approach in our multicentered RCT.^18^^,^^31^ Second, this study only assessed early efficacy and not durability; reassuringly, in our RCT, 90% of medications remained deprescribed at 30 days.^18^ In the 2 prior studies that deployed dialysis-specific deprescribing algorithms, durability was 85% at 6 months^15^ and 77% at 16 months.^44^ Third, although the assignment of units was random, this was a single-center study and was not an RCT. As such, there were slight imbalances in patient comorbid conditions between units. However, the intervention unit had a higher prevalence of some conditions that might have made deprescribing more challenging (eg, diabetic nephropathy, gastroesophageal reflux disorder, and orthostatic hypotension). If anything, we think these imbalances would have biased the intervention toward the null. Fourth, knowledge of an ongoing intervention may have led to the Hawthorne effect.^45^ Nonetheless, nonresearch deprescribing implementation efforts also benefit from clinical champions, as do audit and feedback interventions. Finally, this study was not powered to measure an effect on ADEs, emergency department visits, or hospitalization. We sought to first study whether the process was effective for deprescribing, before running a larger trial. Although deprescribing PIMs is a process measure, it still reduces pill burden for patients and decreases direct drug cost. Whether it also translates to improved outcomes and increased adherence in this population still needs to be demonstrated through a large RCT.

## Conclusion

Deprescribing through clinical decision support in the hemodialysis unit can be effective when paired with the usual medication reconciliation workflow. Future studies will need to evaluate the generalizability and scalability in multiple centers and other countries. Ideally, these studies will have large enough sample sizes to study the effect on ADEs and longer follow-up to evaluate the durability of the intervention.

## Supplementary Materials

Supplementary File (PDF) Figures S1-S3, Items S1-S2.
