# Supplementary material for: Medication Deprescribing in Patients Receiving Hemodialysis: A Prospective Controlled Quality Improvement Study
Source: Kidney Med. 2024 Mar 20;6(5):100810. doi: 10.1016/j.xkme.2024.100810 (PMC11019279; doi:10.1016/j.xkme.2024.100810)
Supplement: Supplementary File (PDF) — Figures S1-S3, Items S1-S2. [file mmc1.pdf]

Supplemental Method 1: Method for capturing gastrointestinal bleeds

Based on a previous, uncontrolled, observational study,[1] there was suspicion that the deprescribing of a proton-pump inhibitor among patients on hemodialysis could increase their risk of developing a gastrointestinal bleed. Although the study we conducted was not sufficiently powered to capture a change in gastrointestinal bleed incidence, the counterbalancing measure for harm was reporting the gastrointestinal bleed occurrences in both the control and intervention units of our study.

The Clinical Informatics Specialist was asked to conduct a search in the electronic medical record's progress notes for the following criteria:

- From September 2022 – April 2023 (beginning of the deprescribing study until 3 months post study completion
- Any patient that had the following word listed in their progress notes during this time:
    - GI bleed ○ UGIB ○ LGIB
    - GIB
    - gastrointestinal bleed ○ red stool ○ black stool ○ hematochezia
    - melena

From these results ÉBC manually extracted the list of patients that were included in the study (both intervention and control units) and reviewed each entry of these patients to determine an occurrence of a gastrointestinal bleed during the above specified timeframe. The study lead would then verify their list of medications and the date of prescription to determine if a proton-pump inhibitor had already been prescribed or not at the time of the gastrointestinal bleed.

Supplemental Method 2: Method for capturing death

From the initial list of patients given to the study lead (control and intervention units), at the end of the study, ÉBC manually conducted a search of each participant's progress notes to capture potential deaths that occurred during the same time frame specified for the reporting of the gastrointestinal bleeds.

Supplemental Figure 1: The Medication Reconciliation Process

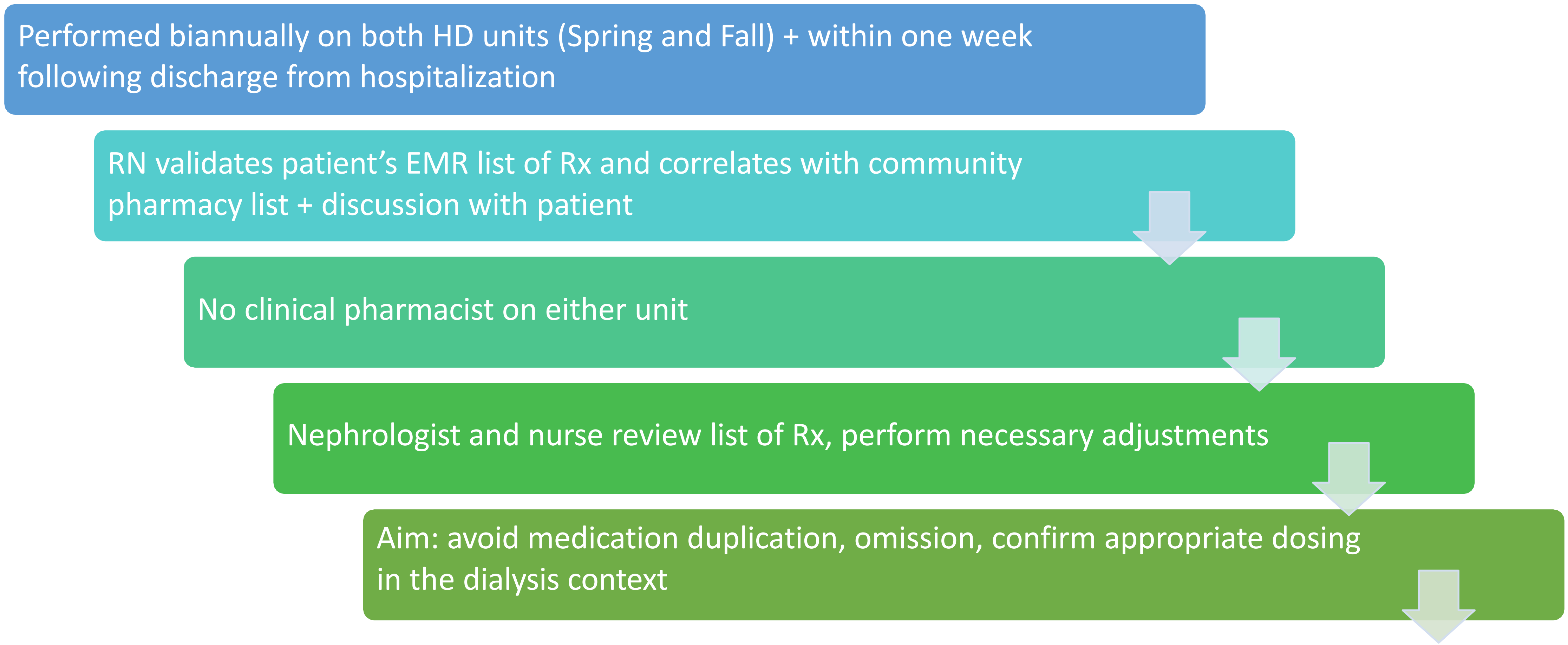

Supplemental Figure 2: Plan Do Study Act (PDSA) Cycles During the Quality Improvement Intervention

PDSA CYCLE 1

- Study lead prepared documentation packages for MedRec eligible patients in advance of team rounds, in alphabetical order.
- This was implemented to improve efficiency.

PDSA CYCLE 2

- Distribution of deprescribing brochures (fact sheet + EMPOWER brochure) to patients only when a relevant drug was deprescribed.
- This was implemented to improve efficiency.

PDSA CYCLE 3

- "MedSafer" was added as a key word to the progress notes following a MedSafer-supplemented MedRec.
- This was implemented to faciliate outcome data collection.

PDSA CYCLE 4

- The study lead emailed the attending nephrologist 1 business day prior to their first day of each rotation with the list of patients requiring a MedRec that week.
- This was implemented to improve efficiency.

PDSA CYCLE 5

- The list of PIMs per patient was also provided to the attending nephrologist, prior to the start of rounding. This built on PDSA cycle 4.
- This was implemented to improve efficiency.

Abbreviations: PDSA=Plan, Do, Study, Act; MedRec=medication reconciliation; PIM=potentially inappropriate medication

Supplemental Figure 3: Subgroup analyses – stratified by age (younger vs. older than 65)

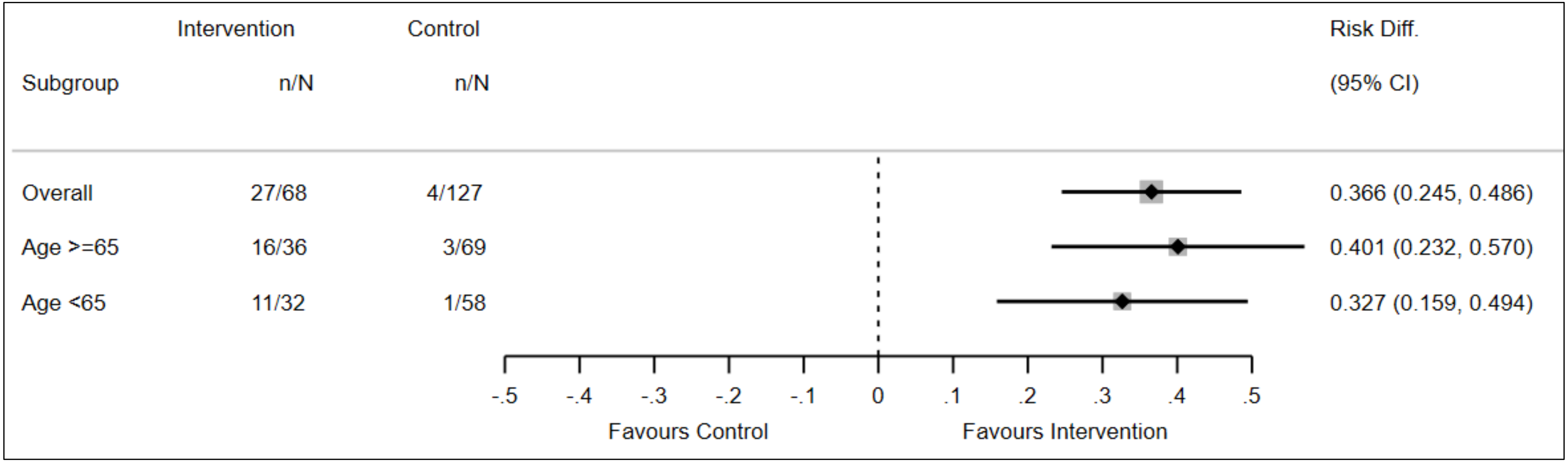

Supplemental Figure 4: Example of a deprescribing EMPOWER brochure

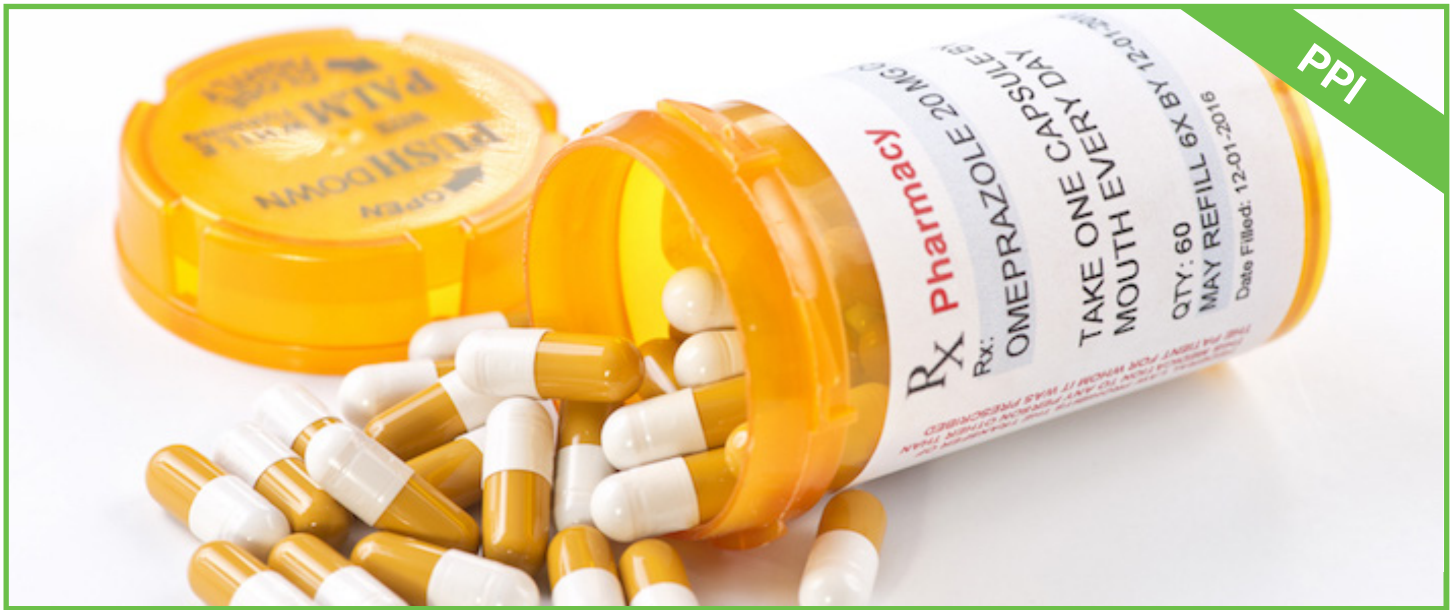

Do I still need this medication?

You are currently taking a proton pump inhibitor (PPI):

- ○ Dexlansoprazole (Dexilant®)
- ○ Esomeprazole (Nexium®)
- ○ Omeprazole (Losec®, Olex®)
- ○ Lansoprazole (Prevacid®, Prevacid Fast Tab®)
- ○ Pantoprazole sodium (Pantoloc®, Panto IV®)
- ○ Pantoprazole magnesium (Tecta®)
- ○ Rabeprazole (Pariet®)

* Generic brands often start with the words: APO, Novo, Pms, Ratio, Sanis, Teva

CIHR IRSC
Canadian Institutes of Health Research | Instituts de recherche en santé du Canada

Centre de recherche iugm
Institut universitaire de gériatrie de Montréal

AFFILIATED WITH Université de Montréal

Michel Saucier Chair in Pharmacy, Health & Aging
La Chaire pharmaceutique Michel-Saucier en santé et vieillissement

Canadian Medication Appropriateness and Deprescribing Network

TEST YOUR KNOWLEDGE ABOUT THIS MEDICATION

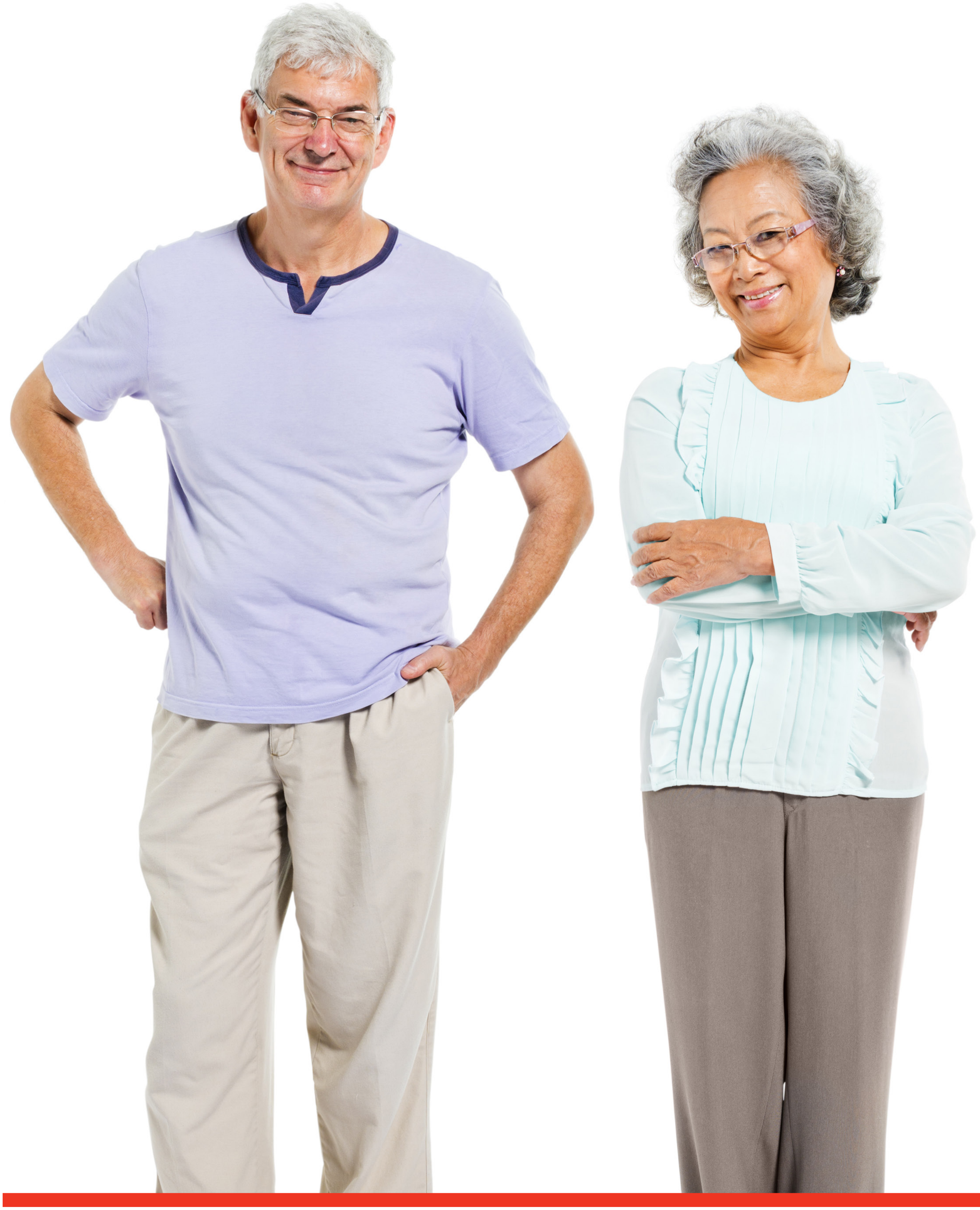

All rights reserved. Copyright © 2014 by Cara Tannenbaum and Institut universitaire de gériatrie de Montréal. Copyright licenses available upon request.

QUIZ

Proton pump inhibitors (PPI)

1. PPIs are sometimes prescribed for heartburn and acid reflux. ☐ TRUE ☐ FALSE
2. More than half of all people taking PPIs probably do not need them. ☐ TRUE ☐ FALSE
3. There are no risks involved in taking PPIs for a long time. ☐ TRUE ☐ FALSE
4. PPIs are the best option to treat occasional heartburn. ☐ TRUE ☐ FALSE

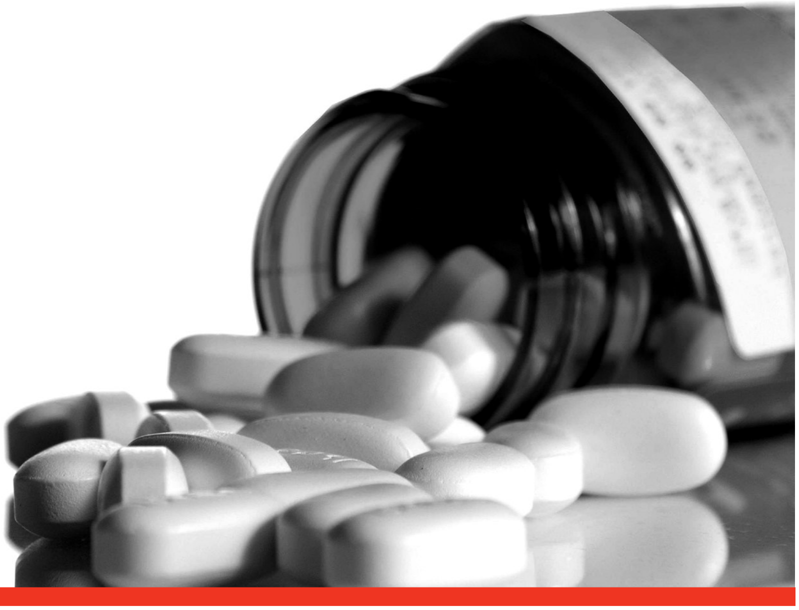

ANSWERS

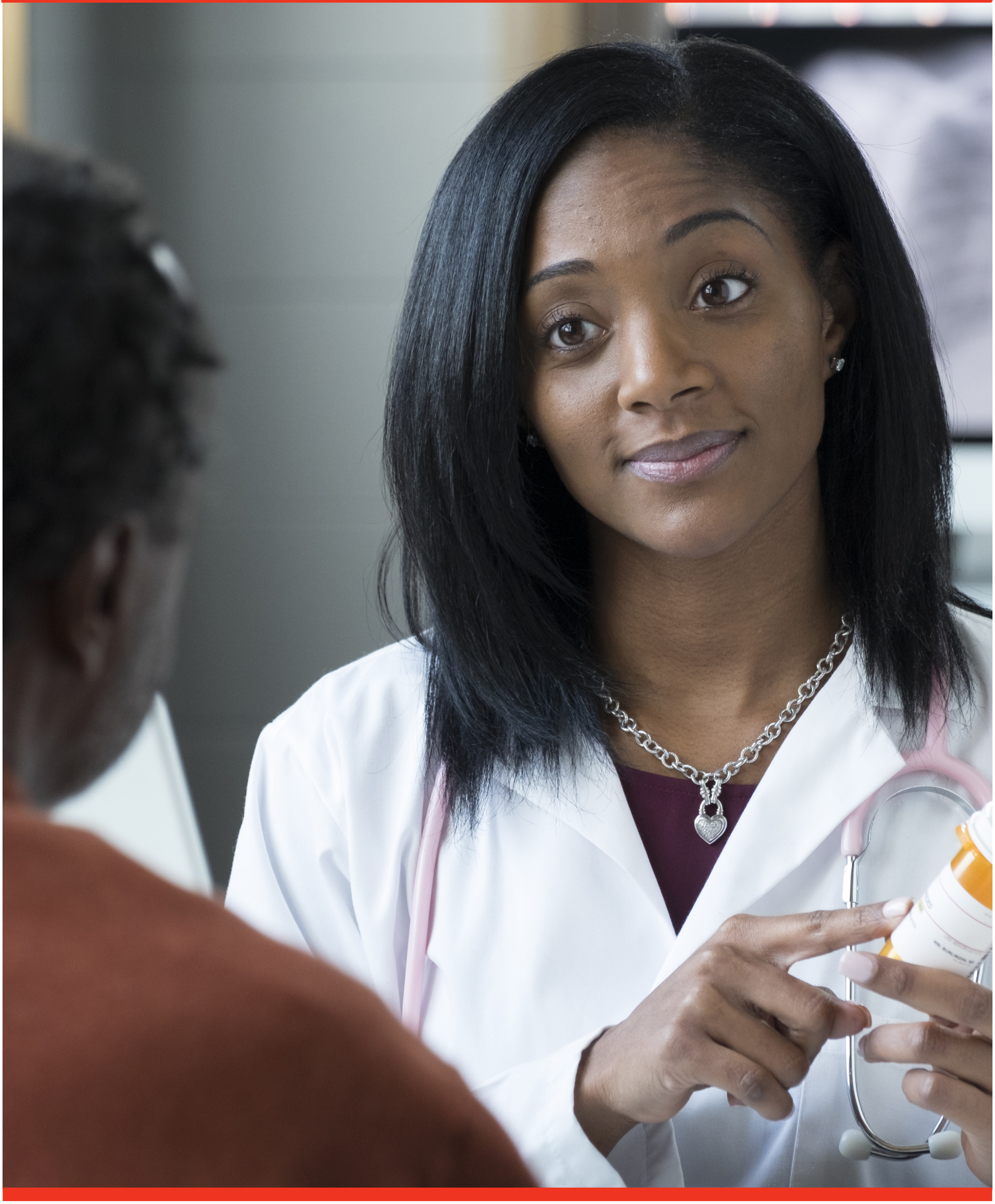

1. TRUE

Proton pump inhibitors (PPIs) are sometimes prescribed to treat heartburn and acid reflux. PPIs reduce the production of acid in the stomach. The stomach produces acid to help break down food, but sometimes the acid can reflux back up the throat and cause discomfort, pain or burning.

2. TRUE

To treat occasional heartburn, it is recommended to take Tums® or Rolaids® as needed. Should your condition require you to take a PPI, your physician should prescribe the lowest dose for the shortest amount of time possible. The next page lists reasons why PPIs should be continued or stopped.

3. FALSE

Taking a PPI for longer than 4 to 12 weeks has been linked to:

- A higher risk of hip fractures
- Pneumonia
- An infection with the bacteria *Clostridium difficile*, which can lead to severe diarrhea, fever, and in rare cases, death
- A higher risk of kidney problems
- Rare instances of vitamin B12 or magnesium deficiency

4. FALSE

PPIs are powerful drugs. If you have heartburn every now and then, you probably do not need a PPI. Over-the-counter antacids should be sufficient. You can ease heartburn without drugs. This brochure explains how.

Do I need to continue taking my PPI?

Check all that apply:

☐ Every day, I take medication that can irritate the stomach, such as anti-inflammatory medication (e.g. ibuprofen or corticosteroids).

☐ I had a major stomach bleed.

If you tick this box, speak to your doctor about your specific circumstances.

I was referred to a gastroenterologist, who looked down my throat with a camera and diagnosed me with:

☐ Barrett's esophagitis.

☐ Severe erosive esophagitis.

If you checked any of these statements, then long-term use of PPIs is usually recommended.

If you don't know the answers, you should talk to your doctor before stopping your PPI.

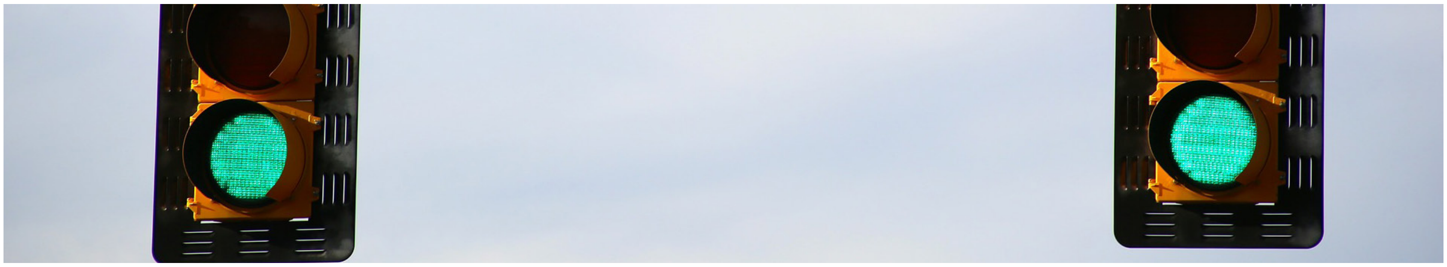

When you need a PPI, you should take the lowest dose for the shortest amount of time possible.

Do I need to stop taking my PPI?

Check all that apply:

- [ ] I no longer have heartburn.
- [ ] My symptoms are infrequent.
- [ ] I have been taking my PPI for longer than 12 weeks and I did not check any of the statements on the previous page (page 6).

If you checked any of these statements, continue reading about how to stop your PPI.

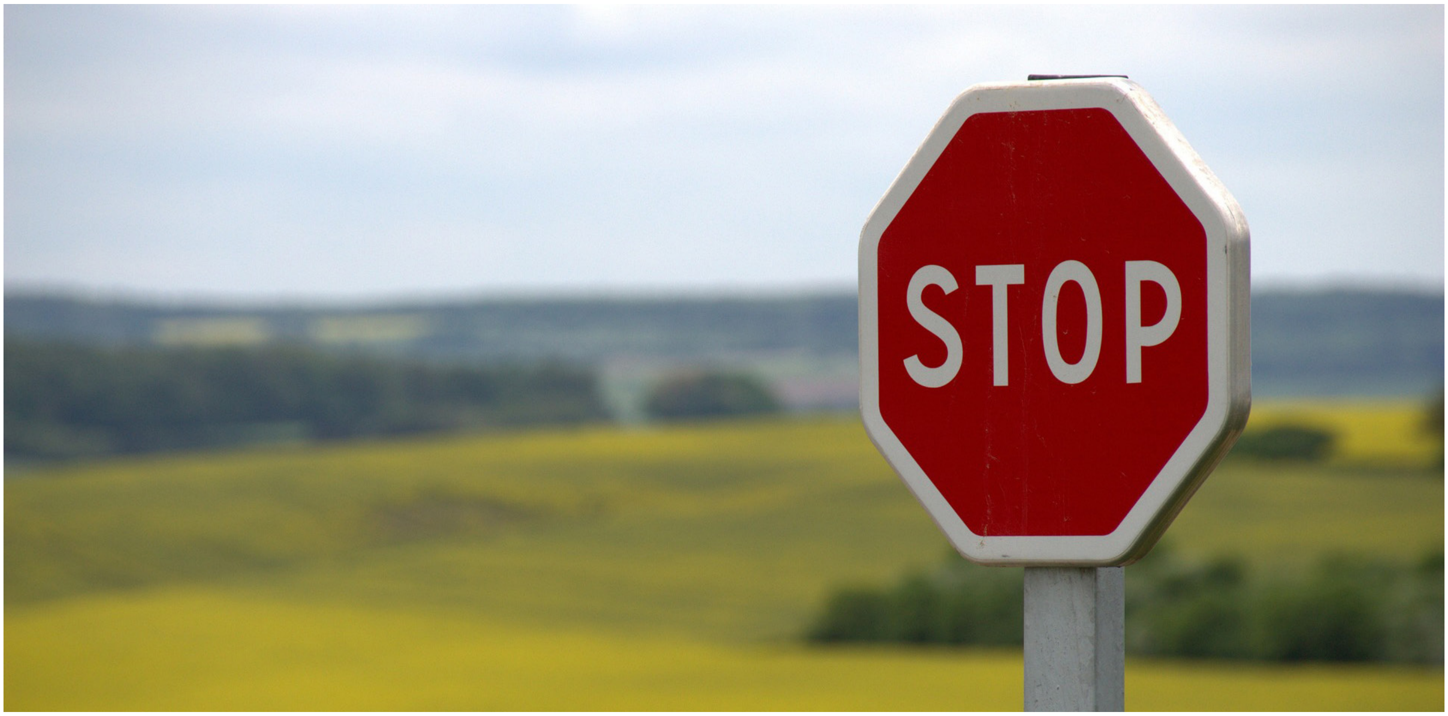

Please consult your doctor, nurse or pharmacist before stopping any medication.

ALTERNATIVES

If you do not need to continue taking PPIs, speak to your doctor, nurse or pharmacist. You can make simple changes in your diet and lifestyle. To prevent heartburn, try these alternatives instead of taking a PPI:

- **Watch what you eat**. Try to figure out which food or beverage triggers your heartburn. You might want to avoid:
  - Alcohol
  - Fried food or junk food
  - Spicy food
  - Garlic and onions
  - Citrus fruits
  - Chocolate and peppermint
  - Food with lots of tomatoes
- **Eat smaller meals.**
- **Do not eat before going to bed.** You could also lie with your head raised up by using extra pillows.
- **Stop smoking.** Studies show that smoking increases your risk of heartburn and acid reflux.
- **Lose weight.** Studies show that just by dropping a few pounds, you could reduce heartburn and acid reflux.
- **Do not wear tight clothes.** The added pressure from tight-fitting clothes that constrict your abdomen can make heartburn worse.

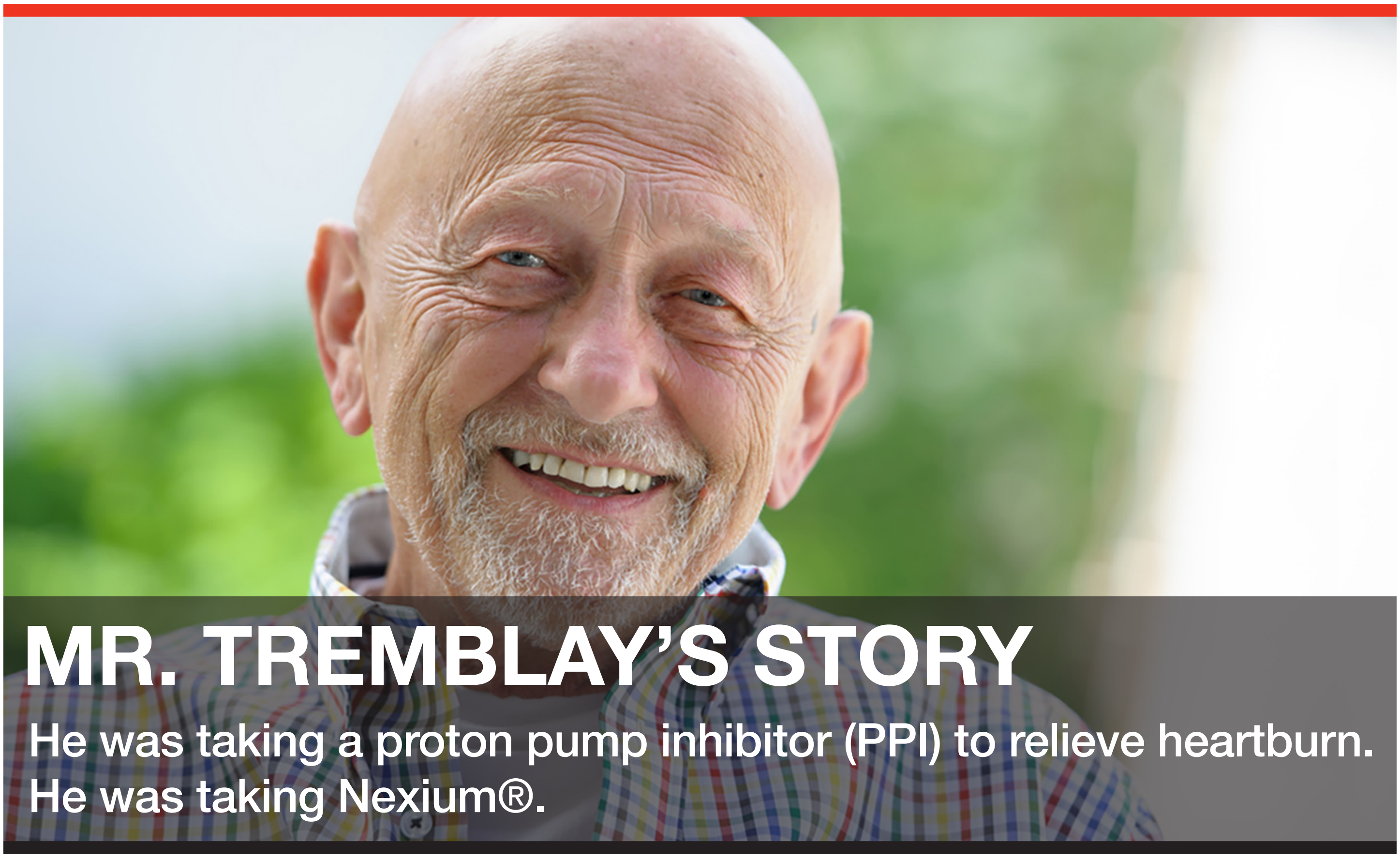

MR. TREMBLAY’S STORY

He was taking a proton pump inhibitor (PPI) to relieve heartburn. He was taking Nexium®.

“I am 65 years old and had been taking Nexium® for at least two years to relieve heartburn. Recently, I was hospitalized for pneumonia. At my next medical visit, my doctor suggested I stop taking Nexium®, as new guidelines show that taking a PPI for more than eight weeks could be linked to pneumonia. Furthermore, my doctor told me it could also interfere with the osteoporosis drug I am now taking.

I took his advice. Now when I get heartburn every now and then, I take Tums® and it does the job.

I also made lifestyle changes. I stopped smoking and I lost a few pounds. Not only did my heartburn almost disappear, but these changes are having a very positive impact on my overall health.

When I know I will be having a big meal, I try to avoid foods that can cause heartburn. I do not drink coffee, I limit my consumption of alcohol and I go out for a walk after dinner.

I know PPIs, like Nexium®, are powerful drugs that have side effects. I trust my doctor to prescribe them only when appropriate and at the smallest dose possible.”

TAPERING-OFF PROGRAM

If you have been taking PPIs for a while, your stomach is probably used to their effect. For some people, suddenly stopping PPIs can lead to rebound acidity and worsening symptoms for a couple of weeks. To minimise these symptoms, it is recommended to slowly taper PPIs over four weeks prior to stopping.

There are 3 approaches that are equally effective in preventing symptom return when you stop your PPI:

1. One approach is to ask your physician to write a new prescription for only half the dose and take this for four weeks, then stop.

2. Alternatively, you can simply skip a pill every second day for four weeks, then stop.

WEEKS	TAPERING SCHEDULE							✓
	MO	TU	WE	TH	FR	SA	SU	
1		●		●		●		
2	●		●		●		●	
3		●		●		●		
4	●		●		●		●	

3. Or, you can use your PPI or alternatives such as ranitidine (Zantac®) or antacids including Tums®, Rolaids® or Maalox® to keep control of your symptoms, only when needed.

In order to select the best option for you, make sure you discuss this with your doctor, nurse or pharmacist.

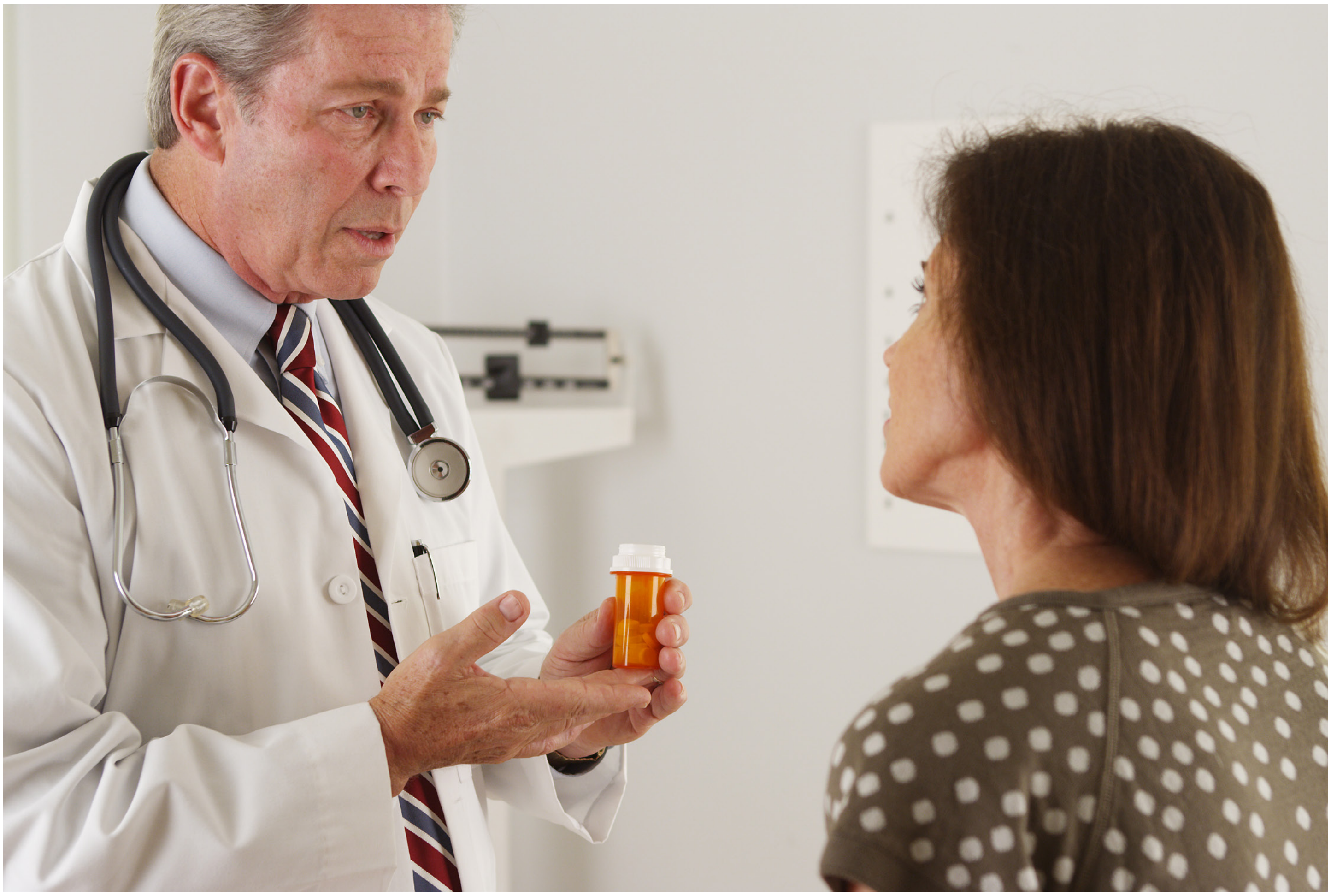

Most PPI tablets or capsules cannot be cut. Please talk with your phamacist before cutting your PPIs.

Make a special appointment to review your medications with your doctor, nurse or pharmacist.

Consult with a health care professional before deciding to taper off your PPIs. You may be on other medications (e.g. anti-inflammatory drugs or corticosteroids), which require you to stay on PPIs or switch to another stomach protective agent.

5 QUESTIONS TO ASK YOUR HEALTH CARE PROVIDER

1. Do I need to continue my medication?
2. How do I reduce my dose?
3. Is there an alternative treatment?
4. What symptoms should I look for when I stop my medication?
5. With whom do I follow up and when?

Questions I want to ask my health care provider about my medication

Use this space to write down questions you may want to ask:

This brochure can be found online at:

www.deprescribingnetwork.ca/useful-resources

All rights reserved. Copyright © 2014 by Cara Tannenbaum and Institut universitaire de gériatrie de Montréal. Copyright licenses available upon request.

Supplemental Figure 5: Example of a MedSafer deprescribing report

MedSafer
Working Towards Safer Prescribing

MedSafer Report - Deprescribing Opportunities

as of 2024-04-04

Mr. Potato Head Male 94yo - Private office

This document contains prioritized **opportunities** for a **reassessment** of the listed medications. Any decisions should take into context what you know about your patient and your clinical assessment of the **risks** and **benefits** of what has been presented.

If you have questions, suggestions, or you would like to report an error, please email support@medsafer.org with the subject line "MedSafer Report"

Tapering instructions or withdrawal concerns?

Please refer to the tapering instructions on last page.

1 Drugs considered **high risk** for adverse drug events

#	DRUG	CAUSE OF ALERT	WHY MIGHT THIS BE INAPPROPRIATE?	TAPERING INSTRUCTIONS
1	clopidogrel (clopidogrel bisulfate) (Plavix)	warfarin sodium (Coumadin)	Dual antithrombotic therapy increases the risk of major hemorrhage. Use of proton pump inhibitor reduces but does not eliminate GI bleeding risk. Dual therapy should be reevaluated for ongoing necessity.	No
2	insulin lispro (Admelog)	Diabetes	Your patient had a recent hemoglobin A1c measurement of less than 7.5%. Avoid using medications known to cause hypoglycemia. In many adults aged 65 and older, who are frail, or have a reduced life expectancy, moderate control (A1c 8-8.5%) is reasonable. Consider decreasing insulin dose.	Yes
3	hydromorphone hydrochloride (Hydromorph.ir)	General	Don’t initiate or maintain opioids long-term for chronic pain until there has been a trial of non-pharmacologic treatment and of non-opioid medications. Non-pharmacologic modalities for chronic pain include exercise, weight loss, cognitive-behavioural therapy, massage and physical therapy. Depending on the pain mechanism and co-morbidities, non-opioid medications include: acetaminophen, NSAIDS and other molecules. An opioid trial should be guided by clear criteria for monitoring the success and a plan for stopping if criteria are not met. This message is adapted from Choosing Wisely Canada.	Yes
4	zopiclone (Zoclone)	General	Don't use benzodiazepines or other sedative-hypnotics in older adults as first choice for insomnia, agitation or delirium.<br><br>For patient material related to this class of medications see link below.	Yes

2 Drugs considered **intermediate risk** for adverse drug event

#	DRUG	CAUSE OF ALERT	WHY MIGHT THIS BE INAPPROPRIATE?	TAPERING INSTRUCTIONS
1	candesartan cilexetil (Atacand)	Hyperkalemia	Risk of precipitating recurrent hyperkalemia, avoid combining with potassium sparing diuretics such as spironolactone if there is a history of hyperkalemia.	No

2	pantoprazole (pantoprazole sodium)<br>(Panto-Byk)	General	Chronic PPI therapy should be reevaluated regularly. For patients aged 60 years and older along with two or more of the following, ongoing therapy may be beneficial: antiplatelet, NSAID, systemic steroids, anticoagulation, prior upper gastrointestinal bleed. Other scenarios requiring ongoing therapy include: hypersecretory conditions, dual antiplatelet therapy, variceal banding within 14 days, and H. Pylori treatment.<br><br>For patient material related to this class of medications see link below.	Yes
3	warfarin sodium<br>(Coumadin)	Dialysis	Consider deprescribing coumadin in patients with Atrial fibrillation and receiving hemodialysis. Apixaban could be a potential alternative, but this remains controversial. Studies are ongoing about the safety of replacing coumadin with apixaban. Prescriber must use clinical judgement in continuing coumadin. Please see guideline on Canadian Cardiovascular Society. Canadian Cardiovascular Society Guidelines for Atrial Fibrillation	Yes

3 Drugs of potentially little benefit or value

#	DRUG	CAUSE OF ALERT	WHY MIGHT THIS BE INAPPROPRIATE?	TAPERING INSTRUCTIONS
1	allopurinol<br>(Alloprin)	Dialysis	If last gout attack was > 12 months ago AND absence of clinical manifestations of gout, initiate deprescribing trial of urate-lowering agent.	Yes
2	docusate calcium<br>(Albert Docusate)	General	Don't use stool softeners to prevent or treat constipation. Controlled studies have shown that drugs such as docusate are ineffective at treating or preventing constipation.	No

Tapering Instructions

NOTE #	DRUG	INSTRUCTIONS
1	**ADMELOG INSULIN LISPRO**	No tapering required. Watch for rebound hyperglycemia.
1	**ALLOPRIN ALLOPURINOL**	No tapering required
1	**HYDROMORPH.IR HYDROMORPHONE HYDROCHLORIDE**	Withdrawal symptoms peak at 48-72 hours and resolve in days to weeks; psychological symptoms may last months. Initial signs: anxiety, sweating, lacrimation, yawning, rhinorrhea, piloerection, anorexia, irritability, mydriasis, cravings. Later signs: insomnia, gastrointestinal upset, tachycardia, hypertension, muscle spasms and pain.<br><br>RECOMMENDATION: Withdraw gradually (empirical decrease by 10% every 5-7 days) and adjust depending on tolerance.<br><br>For patient material on opioids/narcotics: http://www.criugm.qc.ca/fichier/pdf/OpioidsEN.pdf<br><br>Opioid resources from the College of Family Physicians of Canada: https://www.cfpc.ca/chronic-non-cancer-pain-management-opioid-resources/
1	**ZOCLONE ZOPICLONE**	If used daily for more than 3 to 4 weeks, taper more slowly. Decrease by 10 to 25% every 2 weeks. Frequency of dose reductions may need to slow down at smaller doses (ex. 25% of original dose). Monitor every 1 to 2 weeks for duration of tapering. If intolerable symptoms of insomnia occur (usually 1 to 3 days after a dose change), go back to the previously tolerated dose until symptoms resolve, and plan for a more gradual taper.<br><br>For patient material and a tapering regimen with patient/caregiver involvement, please see the following link on sedative-hypnotics: http://www.criugm.qc.ca/fichier/pdf/BENZOeng.pdf
1	**PANTO-BYK PANTOPRAZOLE (PANTOPRAZOLE SODIUM)**	Stopping a PPI abruptly may lead to rebound hyperacidity. Patients on long-term therapy (>3 months) or high dose may benefit from tapering over 2-4 weeks. As needed H2 blocker therapy may mitigate some symptoms of rebound hyperacidity.<br><br>For patient material and a tapering regimen with patient/caregiver involvement, please see the following link on PPIs: http://www.criugm.qc.ca/fichier/pdf/PPI-EN-Men.pdf
1	**COUMADIN WARFARIN SODIUM**	No tapering required

References

1. Czikk D, Parpia Y, Roberts K, et al. De-Prescribing Proton Pump Inhibitors in Patients With End Stage Kidney Disease: A Quality Improvement Project. *Canadian Journal of Kidney Health and Disease* 2022;9:205435812211062. doi: 10.1177/20543581221106244
